# A Multi-Locus Genetic Risk Score for Primary Open-Angle Glaucoma (POAG) Variants Is Associated with POAG Risk in a Mediterranean Population: Inverse Correlations with Plasma Vitamin C and E Concentrations

**DOI:** 10.3390/ijms18112302

**Published:** 2017-11-01

**Authors:** Vicente Zanon-Moreno, Carolina Ortega-Azorin, Eva M. Asensio-Marquez, Jose J. Garcia-Medina, Maria D. Pinazo-Duran, Oscar Coltell, Jose M. Ordovas, Dolores Corella

**Affiliations:** 1 Genetic & Molecular Epidemiology Unit, Department of Preventive Medicine & Public Health, School of Medicine, University of Valencia, Avenida Vicente Blasco Ibáñez 15, 46010 Valencia, Spain; carolina.ortega@uv.es (C.O.-A.); eva.m.asensio@uv.es (E.M.A.-M.); 2 CIBER Fisiopatología de la Obesidad y Nutrición, Instituto de Salud Carlos III, Calle Sinesio Delgado, 3, 28029 Madrid, Spain; oscar.coltell@uji.es; 3 Ophthalmology Research Unit “Santiago Grisolia”, Dr. Peset University Hospital, Avenida Gaspar Aguilar 90, 46017 Valencia, Spain; dolores.pinazo@uv.es; 4 Red Temática de Investigación Cooperativa OftaRed, Instituto de Salud Carlos III, Calle Sinesio Delgado, 3, 28029 Madrid, Spain; josegarciam@yahoo.com; 5 Department of Ophthalmology, University of Murcia, Campus Universitario de Espinardo Building No. 35, 30100 Murcia, Spain; 6 Department of Ophthalmology, Reina Sofia University General Hospital, Avenida Intendente Jorge Palacios, 1, 30003 Murcia, Spain; 7 Department of Surgery, School of Medicine, University of Valencia, Avenida Vicente Blasco Ibáñez, 15, 46010 Valencia, Spain; 8 Department of Computer Languages and Systems, School of Technology and Experimental Sciences, Universitat Jaume I, Avenida Vicent Sos Baynat s/n, 12071 Castellón, Spain; 9 Nutrition and Genomics Laboratory, JM-USDA Human Nutrition Research Center on Aging at Tufts University, 711 Washington Street, Boston, MA 02111, USA; jose.ordovas@tufts.edu; 10 Department of Cardiovascular Epidemiology and Population Genetics, Centro Nacional de Investigaciones Cardiovasculares (CNIC), Calle Melchor Fernández Almagro, 3, 28029 Madrid, Spain; 11 Instituto Madrileño de Estudios Avanzados (IMDEA) Alimentación, Carretera de Canto Blanco 8-E, 28049 Madrid, Spain

**Keywords:** primary open-angle glaucoma, genetics, GWAS, nutrition, vitamin C, vitamin E, genetic risk score

## Abstract

Primary open-angle glaucoma (POAG) is a leading cause of blindness worldwide. The genetics of POAG are complex, and population-specific effects have been reported. Although many polymorphisms associated with POAG risk have been reported, few studies have analyzed their additive effects. We investigated, in a southern European Mediterranean population, the association between relevant POAG polymorphisms, identified by initial genome-wide association studies (GWASs) and POAG risk, both separately and as an aggregated multi-locus genetic risk score (GRS). Also, bearing in mind that oxidative stress is a factor increasingly recognized in the pathogenesis of POAG, we analyzed the potential association of the GRS with plasma concentrations of antioxidant vitamins (C and E). We carried out a case–control study including 391 POAG cases and 383 healthy controls, and analyzed four genetic polymorphisms (rs4656461-*TMCO1*, rs4236601-*CAV1*/*CAV2*, rs2157719-*CDKN2B-AS1* and rs3088440-*CDKN2A*). An unweighted GRS including the four non-linked polymorphisms was constructed. A strong association between the GRS and POAG risk was found. When three categories of the GRS were considered, subjects in the top category of the GRS were 2.92 (95% confidence interval (CI): 1.79–4.77) times more likely to have POAG compared with participants in the bottom category (*p* < 0.001). Moreover, the GRS was inversely correlated with plasma vitamin C (*p* = 0.002) and vitamin E (*p* = 0.001) concentrations, even after additional adjustment for POAG status. In conclusion, we have found a strong association between the GRS and POAG risk in this Mediterranean population. While the additional correlation found between GRS and low levels of vitamins C and E does not indicated a causal relationship, it does suggest the need for new and deeper research into the effects of oxidative stress as a potential mechanism for those associations.

## 1. Introduction

Primary open-angle glaucoma (POAG) is the most common form of glaucoma. It is a form of optic neuropathy characterized by high intraocular pressure (IOP), an alteration of the optic nerve head, and a loss of visual field [1]. POAG is one of the main causes of blindness worldwide, and currently has no cure. One of the most important risk factors for POAG is IOP. Therefore, medical treatment, including both eye drops and surgery, is focused on halting the progression of this disease, thus preventing bilateral blindness by means of ocular tension control [2]. However, POAG is a multifactorial disease that involves both genetic and environmental factors (including diet), so the development of the glaucomatous disease is determined not only by the individual effect of each of these factors, but also by the joint effect of the interaction between all of them [3,4].

Many studies have been carried out in order to advance our knowledge of the genetic bases of POAG (reviewed in [5]). Thus, several polymorphisms in candidate genes have been associated with the risk of this type of optic neuropathy [6,7,8]. Likewise, initial genome-wide association studies (GWASs), have also identified common variants in several genes at high risk for this disease. However, despite the identification of several novel variants by the initial GWAS, these variants may not be universal risk factors in all populations [5]. One of the POAG-variants reported in the initial GWAS in Caucasian populations was the rs4656461 polymorphism in the transmembrane and coiled-coil domains 1 gene (*TMCO1*) [9,10]. This gene is expressed in several human tissues [10,11]. Although *TMCO1* plays a role in tumor suppression and cell cycle regulation [9], the function of the protein encoded by this gene in relation to POAG is still unknown. Another initial GWAS has also associated common variants near the caveolin 1 (*CAV1*) and caveolin 2 (*CAV2*) genes with POAG risk [12]. *CAV1* and *CAV2* play an important role in caveolar biogenesis. Both the *CAV1* and *CAV2* genes are expressed in ocular tissues [13] and both genes may play an important role in glaucomatous alterations of trabecular meshwork cells [14]. Oxidative stress-induced dysfunction in trabecular meshwork cells is considered a major alteration that can lead to glaucoma [15]. The trabecular meshwork in POAG is characterized by cellular senescence, increased accumulation of extracellular matrix [16], and other processes that oxidative stress is able to trigger [17]. *CAV1* is also involved in nitric oxide metabolism through its interaction with the endothelial form of nitric oxide synthase (eNOS) which turns eNOS into an inactive form, so decreasing the production of nitric oxide [18]. Moreover, there is increasing evidence that the *CAV1* protein is an oxidative stress-related protein [19,20]. Thus, *CAV1* may be a target of antioxidants in oxidative stress modulation for prevention.

A long non-coding RNA encoded in the chromosome 9p21region, the antisense noncoding RNA in the INK4 locus (ANRIL), has been classically associated with cardiovascular diseases and more recently with cancer, diabetes or glaucoma in an initial GWAS [5,10,21,22]. This locus regulates tumor suppressors cyclin dependent kinase inhibitors 2A and 2B (*CDKN2A* and *CDKN2B-AS1*) through epigenetic mechanisms [23]. *CDKN2A* (also called p16) is a regulator of transcription and apoptosis and is also related to age-dependent modifications of the cell cycle-negative regulation of human cornea endothelial cells [24]. *CDKN2B-AS1* (also called p15) plays a role in the control of G1/S transition in the cell cycle [25].

However, despite the relevance of the initial findings of the new genes in the GWAS, it appears that controversy still exists around determining the clinical utility of the genetic variants discovered, bearing in mind that the association results initially reported were not similar in several subsequent studies carried out in different populations [5]. This potential heterogeneity of the effects, as well as of the allele frequency of the POAG variants, highlight the importance of estimating the specific risk of the most relevant variants in the context of geographic ancestry. For this reason, our aim was to analyze, in the Spanish Mediterranean population (which was not included in previous GWASs), the association of the most relevant polymorphisms discovered in the first GWAS carried out on various populations (including the loci *TMCO1*, *CAV1*/*CAV2* and *CDKN2A* and *CDKN2B-AS1*) [10,12] with POAG risk in this population. It is not only necessary to undertake this investigation with individual Single Nucleotide Polymorphisms (SNPs), but also to study their possibly additive contribution through genetic risk scores (GRSs), as there are few studies in the field of POAG risk that have analyzed GRSs.

Nevertheless, as mentioned above, not only genetic factors are important in the onset and progression of the glaucomatous disease. Nutrition is also essential to maintaining good eye health status [4,26,27]. Diet provides antioxidants to the organism, helping to counteract oxidative stress [28,29] that, as is known, plays a key role in the etiopathology of glaucoma [30,31]. Therefore, a good nutritional status with an adequate intake of antioxidants might result in better prevention and management of this optic neuropathy [4,27,32]. Our group carried out several studies showing the strong association between plasma levels of vitamins and POAG risk [33,34].

Thus, taking into account the high genetic heterogeneity of POAG depending on the populations studied, as well as the lack of knowledge about the potential mechanisms by which genetic variants may influence POAG risk, the aims of this study are: (1) to analyze the association of selected loci (*TMCO1*, *CAV1*/*CAV2*, *CDKN2B-AS1* and *CDKN2A*), obtained from previously published GWAS, with POAG risk in a southern European Mediterranean population; (2) to study the effect of the simultaneous presence of the genetic risk variants, by means of a genetic risk score (GRS), in order to jointly estimate the contribution of these variants to POAG risk in this population; and (3) to investigate the possible correlation between the GRS for POAG risk and plasma levels of antioxidant vitamins (C and E) in this population.

## 2. Materials and Methods

We carried out a case-control study in 391 subjects with POAG (cases) and 383 healthy controls, recruited from the Dr. Peset University Hospital (Valencia, Spain) and the Department of Preventive Medicine and Public Health of the School of Medicine of the University of Valencia (Spain). The Ethical Committee of the University of Valencia approved the protocols for this study, which complied with the Helsinki guidelines on human research. Informed consent forms were signed by all study participants.

The diagnosis of POAG was performed based on three ophthalmic tests: (1) the measurement of ocular tension using a Goldman applanation tonometer; (2) the evaluation of papillary excavation by optical coherence tomography; and (3) the quantitative analysis of visual field by means of computerized perimetry.

Cases were subjects with POAG and ages ranged from 40 to 80 years. All patients had high intraocular pressure (21 mmHg or higher). Controls were subjects without eye diseases and with ages in the same range as glaucomatous patients. Subjects with ocular diseases other than POAG, such as cataracts, age-related macular degeneration, or severe myopia (6 or more diopters), and/or age outside the range of inclusion were excluded.

An ophthalmologic examination was performed on all subjects, both cases and controls, to measure intraocular pressure. Also, cases and controls completed a questionnaire regarding socio-demographic, clinical, and lifestyle variables.

Whole blood samples were collected from each subject (Ethylenediaminetetraacetic acid (EDTA) tubes) under fasting conditions. One of those tubes was used to determine the plasma levels of vitamin C and vitamin E. Another tube was used for DNA isolation and the analysis of the selected genetic polymorphisms.

### 2.1. Determination of Vitamins C and E in Plasma

Plasma vitamin C concentrations were measured using the Li et al. method [35]. Analyses were carried out using Shimadzu Scientific Instruments (SSI, Columbia, MD, USA) equipment with an LC-20AB delivery pump and an electrochemical detector, under reversed-phase conditions with a 4.6 × 250 mm, 5 μM YMC-Pack ODS-AQ column (Waters Corp., Milford, MA, USA). The software used was LabSolutions 1.2 (SSI, Columbia, MD, USA). Compounds were eluted over an 18-min runtime at a flow rate of 0.6 mL/min. The mobile phase consisted of methanol/150 mM chloroacetate (3:97, *v*/*v*) and 2 mM disodium EDTA (pH adjusted to 3.0 with NaOH). Sample injection was 5 μL.

Plasma vitamin E concentrations were determined by the Arnaud et al. method [36] using Shimadzu Scientific Instruments equipment with an LC-20AB delivery pump and a UV-Vis detector (290 nm) with a 4.6 × 250 mm, 5 μM YMC-Pack ODS-AQ column (Waters Corp., Milford, MA, USA). The software used was LabSolutions 1.2 (SSI, Columbia, MD, USA). Compounds were eluted over a 20-min runtime at a flow rate of 1.2 mL/min. The mobile phase consisted of acetonitrile/dichloromethane/methanol (72.5/22.5/5). Sample injection was 50 μL.

### 2.2. Analysis of Genetic Polymorphisms

Genomic DNA was extracted from blood samples using the MagNA Pure LC DNA Isolation Kit (Roche Diagnostics Inc., Indianapolis, IN, USA). We have focused on the more relevant genes associated with POAG risk in the first GWASs undertaken in Caucasian populations [10,12]. Those GWASs found SNPs statistically significant at the genome-wide level in the regions of genes *CAV1*/*CAV2*, *TMCO1* and *CDKN2A*/*CDKN2B-AS1*. These loci were selected precisely because, despite being the first genes identified, controversy involving replication in different populations still exists. The selected SNPs were preferably those that showed a greater association in the initial GWASs and those that were not in linkage disequilibrium with each other, in order to maintain independence (r^2^ < 0.2) as a general rule for building the GRSs. The genotyping of four polymorphisms selected from GWAS studies was performed by means of the TaqMan fluorescent allelic discrimination technique, using a real-time Thermal Cycler (7900HT Sequence Detection System, Applied Biosystems, Carlsbad, CA, USA). The SNPs analyzed were: rs4656461 in the *TMCO1* gene (Applied Biosystems, ref. C___1790879_20), rs4236601 near the *CAV1* and *CAV2* genes (Applied Biosystems, ref. C__26498900_10), rs2157719 in the *CDKN2B-AS1* gene (Applied Biosystems, ref. C___2618013_10) and rs3088440 in the *CDKN2A* gene (Applied Biosystems, ref. C__16008027_10).

### 2.3. Statistical Analyses

Qualitative variables were compared using the chi-square test. The Hardy–Weinberg equilibrium for genotype frequencies in controls was also checked using this test. Two means were compared using Student's t-test, and more than two means were compared using the ANOVA test. Multivariable general linear models were used to adjust differences in continuous variables by potential confounders (sex, age, or POAG status, depending on the model) as well as to estimate the corresponding adjusted means. Logistic regression analyses were used to estimate the risk of POAG by means of the odds ratio (OR) and the corresponding 95% confidence intervals (CI). Unadjusted and multivariable adjusted models were fitted. These models were adjusted for age and gender depending on the analysis, as indicated.

An unweighted GRS (uGRS) was calculated by the sum of the risk alleles (0, 1 or 2) for all the non-linked SNPs (r^2^ < 0.2) entering in the combined score (*TMCO1*-rs4656461, *CAV1*/*CAV2*-rs4236601, *CDKN2B-AS1*-rs2157719 and *CDKN2A*-rs3088440). The GRS was first considered as a continuous variable and then categorized into a three-category variable: (1) low (0 or 1 points); (2) medium (2 or 3 points); and (3) high (4, 5, 6 or 7 points).

We also calculated the weighted genetic risk score (wGRS) by multiplying the number of risk alleles of each Single Nucleotide Polymorphisms (SNP) by the corresponding size effects in this population (the corresponding OR per risk allele for each SNP in the separate association analysis, adjusted for age and sex) and summing the products.

Finally, a receiver-operating characteristics (ROC) curve analysis was performed in order to calculate the area under the curve (AUC) to assess the discriminatory ability of the uGRS and wGRS to predict POAG. We used IBM SPSS Statistics software (version 24.0, IBM Corporation 2017, Armonk, NY, USA) for statistical analyses of data.

## 3. Results

We analyzed 391 POAG cases and 383 controls. Socio-demographic and clinical characteristics of cases and controls are shown in Table 1.

**Figure 1:**
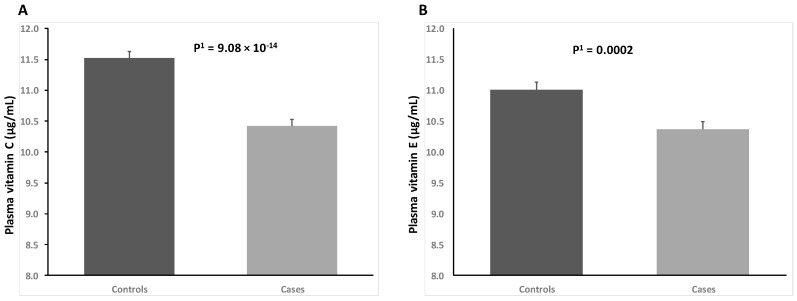
Plasma concentrations of vitamin C (panel **A**) and vitamin E (panel **B**) in POAG cases and controls. P^1^ denotes the *p*-value for the comparison of unadjusted means between cases and controls. Error bars show the standard error (SE) of means.

Plasma vitamin C and vitamin E concentration were significantly lower in POAG cases as compared to controls (Figure 1). These differences remained statistically significant after additional adjustment for sex and age, with adjusted means and standard error (SE) of plasma vitamin C: 11.61 (0.11) μg/mL in controls versus 10.41 (0.11) μg/mL in POAG cases (*p* < 0.001) and adjusted means of plasma vitamin E: 11.00 (0.12) μg/mL in controls versus 10.39 (0.12) μg/mL in POAG cases (*p* < 0.001).

The genotype frequencies for all SNPs analyzed were in Hardy–Weinberg equilibrium in controls (*p* = 0.102, *p* = 0.961, *p* = 0.055 and *p* = 0.946 for the SNPs in *TMCO1*, *CAV1*/*CAV2*, *CDKN2B-AS1* and *CDKN2A*, respectively). The crude statistical analysis of genotype frequencies of the polymorphisms studied showed a significant association of the SNPs in the *TMCO1* gene, *CAV1*/*CAV2* loci, and *CDKN2B-AS1* gene with the risk for POAG (Table 2). The *CDKN2A*-rs3088440 polymorphism was also significantly associated with POAG risk after multivariate adjustment for sex and age. The strongest association was found for the rs4656461-*TMCO1* (A>G) SNP. For this SNP, heterozygous subjects for the risk allele (G) presented an OR: 1.44; 95% CI: 1.05–1.96 in comparison with homozygous subjects (AA) for the non-risk (major) allele. Likewise, POAG risk for homozygous subjects for the minor allele (G) was OR: 2.03; 95% CI: 1.17–3.52) in comparison with AA individuals. Additional adjustment for sex and age did not change the statistical significance of results.

We first calculated the uGRS, as described in Materials and Methods, including the rs4656461-*TMCO1*, rs4236601-*CAV1*/*CAV2*, the rs2157719-*CDKN2B-AS1* and the rs3088440-*CDKN2A* polymorphisms. The mean value (±SD) of the uGRS as a continuous variable in POAG patients (uGRS: 2.60 ± 1.19) was significantly higher (*p* = 0.00005) than that for control subjects (2.17 ± 1.32). Supplemental Figure S1 shows the GRS distribution in POAG cases and controls. In the logistic regression analysis, a 34% increased risk of POAG per risk allele was found for the uGRS as a continuous variable (OR: 1.34 per risk allele; 95% CI: 1.17–1.48; *p* = 0.00007). This association remained statistically significant after adjustment for age and sex (OR: 1.38 per risk allele; 95% CI: 1.22–1.57; *p* = 3.8 × 10^−7^).

Additionally, three categories for the uGRS were considered, as detailed in Materials and Methods. Table 3 shows the association between the categorical uGRS and POAG risk in the whole population. This uGRS was strongly associated with POAG risk. Subjects in the highest category of the score have a 2.92-fold increased risk of POAG as compared with those in the lowest category (*p* < 0.001) in the model adjusted for age and sex.

**Figure 2:**
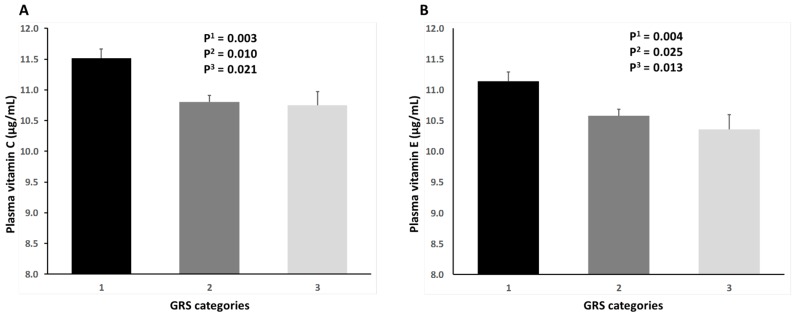
Plasma concentrations of vitamin C (panel **A**) and vitamin E (panel **B**) depending on the genetic risk score (GRS) as a categorical variable: category 1 (0–1 risk alleles); category 2 (2–3 risk alleles); and category 3 (4–8 risk alleles). P^1^: *p*-value for the unadjusted comparison of means. P^2^: *p*-value obtained in the model adjusted for the POAG status. P^3^: *p*-value obtained in the model adjusted for POAG status, age and sex. Error bars are SE of means.

We also analyzed the statistical association between the categorical uGRS and plasma levels of vitamin C and vitamin E (Figure 2). For vitamin C (Figure 2A) we found a statistically significant association in the unadjusted model, in such a way that subjects in the lowest category of the uGRS had significantly higher plasma vitamin C concentrations than subjects in the top category of the uGRS (*p*-trend = 0.003). This inverse correlation may reflect the lowest plasma vitamin C levels in POAG cases. However, it remained statistically significant even after adjustment for POAG status, age and sex (adjusted *p*-value = 0.021). Nevertheless, these results should be interpreted with caution taking into account that we did not carry out a formal mediation analysis and this statistical correlation did not indicate causality. Likewise, for plasma vitamin E concentrations (Figure 2B), mean differences among the three categories of the uGRS reached statistical significance in the unadjusted model (*p*-trend = 0.004) and the differences (highest levels in subjects in the lowest GRS category) remained statistically significant in the model adjusted for POAG status, age and sex (*p* = 0.013). 

Similar correlation results were obtained when the uGRS was used as a continuous variable. Table 4 shows regression coefficients (B) between the uGRS (as continuous) and plasma concentrations of vitamin C and E in the different regression models. Even after multivariable adjustment for POAG status, age and sex, both for vitamin C and vitamin E, a higher uGRS was inversely associated with lower plasma vitamin concentrations (B = −0.186 μg/mL of plasma vitamin C per POAG risk allele; *p* = 0.004 in and B = −0.233 μg/mL of plasma vitamin E per POAG risk allele; *p* = 0.001). These inverse correlations did not indicate causality.

Furthermore, we also calculated a wGRS including the same polymorphisms. Similar association results were obtained for the wGRS to those obtained with the uGRS. The mean value (±SD) of the continuous wGRS in POAG patients (wGRS: 3.65 ± 1.69 points) was significantly higher (*p* = 0.00005) than that (3.05 ± 1.88 points) in the control subjects. This difference remained statistically significant after adjustment for age and sex (*p* = 1.97 × 10^−7^). As a continuous variable, in the logistic regression model, the wGRS was significantly associated with POAG risk (OR: 1.21; 95% CI: 1.11–1.32; *p* = 0.000008). Likewise, the wGRS was also significantly associated with plasma concentrations of vitamin C and vitamin E. After multivariable adjustment for POAG status, age and sex, both for vitamin C and vitamin E, a higher wGRS was inversely associated with lower plasma vitamin concentrations (B = −0.128 μg/mL of plasma vitamin C per point in the score; *p* = 0.005 in and B = −0.172 μg/mL of plasma vitamin E per point in the score; *p* < 0.001). These results were similar to those obtained for the uGRS.

**Figure 3:**
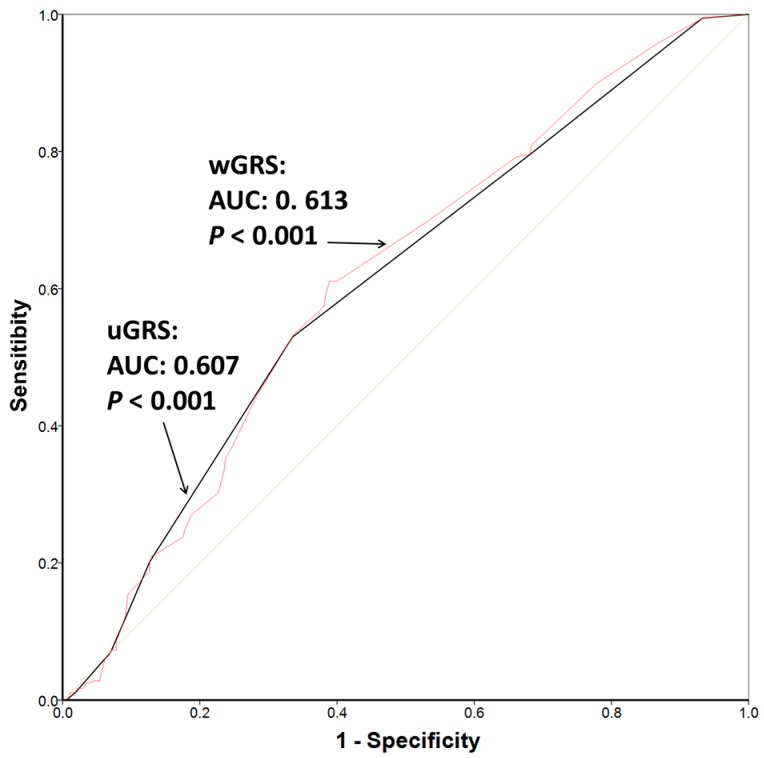
Receiver operating curves (ROCs) of the two (unweighted and weighted) genetic risk scores (GRSs) to predict POAG risk in the 391 cases and 383 controls. Both GRSs included the POAG-SNPs: *TMCO1*-rs4656461; *CAV1*/*CAV2*-rs4236601; *CDKN2B-AS1*-rs2157719; and *CDKN2A*-rs3088440. Area under the curve (AUC) and *p*-values for the corresponding GRS are shown.

Finally, we plotted the ROC curves (Figure 3) and estimated the AUC for the uGRS and the wGRS. Analogous results were found for both scores. The AUC for the uGRS was 0.607 (95% CI: 0.567–0.648; *p* = 5.3 × 10^−7^), and the AUC for the wGRS was 0.613 (95% CI: 0.573–0.654; *p* = 1.2 × 10^−7^). Thus, for reasons of simplicity, we preferred the use of the uGRS for this multi-locus GRS.

## 4. Discussion

POAG is a complex disease [2] showing a high genetic heterogeneity across populations [5,37,38,39,40]. In the present study we have analyzed whether selected POAG SNPs discovered in previous GWASs are associated with POAG risk in a southern European Mediterranean population, obtaining significant associations. Moreover, we have estimated the combined POAG risk of the selected polymorphisms by mean of a multi-locus GRS, revealing higher associations. This provides new information, given that the majority of previous studies did not calculate GRSs for estimating the combined genetic risk. Furthermore, we have found that the POAG GRS is inversely correlated with plasma antioxidant vitamin (C and E) concentrations, thus suggesting the interest of new studies examining this link as a potential mechanism shared by the genes included in the GRS.

Different processes have been related to POAG etiopathogenic mechanisms, such as inflammation, apoptosis, or vascular dysfunction [41,42,43,44]. Oxidative stress has also been associated with glaucoma [30,45,46]. Here, we have expanded on the previous findings of our group, detecting statistically significant lower plasma levels of the antioxidant vitamins C and E in a large number of POAG patients and controls [34]. Goyal et al. [47] reported similar results, showing significantly lower concentrations of both these vitamins in POAG cases and suggesting that high oxidative stress might play a role in the pathogenesis of this optic neuropathy. Ko et al. [48] went a step further and demonstrated, in an experimental model of glaucoma, that vitamin E deficiency could cause an increase of retinal ganglion cell death, which may be associated with the increase of lipid peroxidation.

In addition to these studies on oxidative stress, deepening our knowledge on the genetics and genomics of POAG is another important goal of glaucoma research. However, POAG is a multigenic disease, making it difficult to study. Since the initial association between the myocilin (MYOC) locus and this form of optic neuropathy [49], several other candidate loci have been related to POAG. However, the limited heritability explained by these findings on candidate loci led researchers to carry out GWASs for POAG, as a way of addressing POAG genetic complexity [50,51]. Nevertheless, important heterogeneity in the effects in the discovered POAG risk variants across populations has been noted and specific studies for validation of the associations in different populations are needed to better understand the genetic effects and their applications [39]. We selected four relevant SNPs from initial GWASs to confirm their association with POAG in a southern European Mediterranean population. Among them, we analyzed the rs4656461 polymorphism near the *TMCO1* gene. This SNP was reported by Burdon et al. [10] in a GWAS carried out in participants from Australia and New Zealand. In this GWAS, the rs4656461[G]-*TMCO1* reached the highest significance level (*p* = 6.1× 10^−10^). Thus, homozygous subjects for the G allele of this polymorphism had a high risk of POAG. In the present study, we have replicated the association between this SNP and the risk for POAG in a Mediterranean population, with GG subjects having a 2-fold risk of POAG in comparison with homozygous AA subjects. Although the function of this gene is still unknown, Sharma et al. [9] suggested that it may play a role in tumor suppression and cell cycle regulation. It has been proven that retinal ganglion cell death by apoptosis is a hallmark of POAG [50]. Thus, *TMCO1* may be involved in the mechanisms underlying retinal ganglion cell degeneration. However, the precise functions of the protein and of the common variants in the *TMCO1* gene in relation to POAG still need to be elucidated.

The second SNP most associated with POAG risk in the GWAS carried out by Burdon et al. [10] was the rs4977756-*CDKN2B-AS1* on chromosome 9p21. For this SNP, the major allele was associated with higher POAG risk, mainly for normal pressure glaucoma. Variants in this locus have also been related to the risk for glaucoma in other studies with different results [5,37,38,39]. Wiggs et al. [51], analyzing subjects recruited in the United States, found several variants in the *CDKN2B-AS1* locus, including the rs2157719-*CDKN2B-AS1* polymorphism, with the minor allele being protective against glaucoma. However, in our study (including cases with high pressure glaucoma), the minor allele for the rs2157719 SNP was associated with increased risk of POAG. Taking into account that the Mediterranean and the American populations differ in genetic background, and that population-specific associations have been reported for several POAG polymorphisms [5], this may explain the observed results. Interestingly, in another publication of the same group, this time analyzing the association among the *CDKN2B-AS1* SNPs and glaucoma features among POAG patients [52], they reported that for several of the protective *CDKN2B-AS1* SNPs with minor alleles associated with reduced POAG risk, POAG patients carrying the minor allele had higher IOP. Likewise, the minor allele of the rs2157719-*CDKN2B-AS1* polymorphism has also been associated with increased intraocular pressure in POAG patients from China [53]. In our Mediterranean population, POAG cases had significantly higher IOP than controls, also contributing to an explanation of the results.

We also studied the *CAV1* and *CAV2* loci in chromosome 7, reported in an initial GWAS [12]. These genes are members of the caveolin gene family and are expressed in visual systems, specifically in the trabecular meshwork and retinal ganglion cells [54]. Both the caveolin 1 and caveolin 2 proteins are involved in caveolae formation, plasma membrane invaginations rich in cholesterol and other lipids [12]. For this region, several ethnic-specific associations of the *CAV1*/*CAV2* loci with POAG risk have been reported [39,40]. The analyzed SNP, the rs4236601 (G/A) polymorphism, is located near *CAV1* and *CAV2*. Frequencies of the risk allele (the minor allele) are very different across populations. In Caucasians, the minor allele is present in 20–28% of the control subjects, conferring an odds ratio (OR) of 1.1 to 1.38 in several studies [5,12]. In contrast, the rs4236601 polymorphism is rare in East Asians. In a GWAS study carried out by Thorleifsson [12], the authors found an association between the risk variant of the rs4136601 polymorphism (near *CAV1* and *CAV2* genes) and POAG in Iceland. However, some subsequent studies with participants from the United States (Iowa) [55], Saudi Arabia [56], or Africa [57] have not replicated this association. Interestingly, rs4236601 has recently been significantly associated with higher POAG risk in two Chinese cohorts (the Hong Kong Chinese cohort and the Beijing cohort), but was not polymorphic in a Japanese cohort (the Osaka cohort) [40]. In our southern Mediterranean population, we confirmed the association of this polymorphism with higher POAG risk, supporting the hypothesis that alterations in these genes might play a role in the glaucomatous pathogenesis.

For the rs3088440-*CDKN2A* polymorphism, not statistically linked to the rs2157719-*CDKN2B-AS1* polymorphism located in the region of chromosome 9p21 (*CDKN2A*/B) and widely studied for the association with POAG risk, the published results again have been population-specific [51,58,59,60]. In this Mediterranean population we have found a significant association of the minor allele with higher POAG risk.

In addition to the separate analysis of each polymorphism, we calculated the combined GRS (both unweighted and weighted). Although the use of GRS has been very common for obesity or cardiovascular diseases, in the field of POAG, the calculation of multi-locus GRSs is still very limited and mainly focuses on IOP or other POAG-related phenotypes [61,62]. As far as we know, our study is the first study showing a strong additive association between the four selected polymorphisms and POAG risk. Moreover, we have reported for the first time an inverse correlation between the additive GRS and plasma concentrations of vitamins C and E. Although the polymorphisms studied have not previously been associated with plasma concentrations of the antioxidant vitamins C and E, there is prior evidence that these vitamins are reduced in POAG cases [34,63]. Likewise, as stated before, one factor increasingly argued in the pathogenesis of POAG is oxidative stress [45,46,47]. It has also been shown that antioxidant capacity, measured through various markers including antioxidant vitamins, is reduced in POAG and that low levels of vitamins aggravate the glaucomatous process [41,48]. Therefore, while it is not entirely clear whether the inverse relationship between oxidative stress and POAG is a cause or a consequence, what is clear is that they are very closely related. Although the genes included in the GRS have not previously been related with antioxidant vitamin concentrations, there is some prior evidence of their participation in processes related with oxidative stress. Thus, both *CAV1* and *CAV2* genes are expressed in ocular tissues [13] and both genes may play an important role in glaucomatous alterations of trabecular meshwork cells [14]. Oxidative stress-induced dysfunction in trabecular meshwork cells is considered a major alteration that can lead to glaucoma [16]. Besides these effects at the ocular level, it has generally been shown that the caveolin-1 protein inhibits expression of antioxidant enzymes through direct interaction with nuclear erythroid 2 p45-related factor-2 (Nrf2) [19], thus having a more systemic implication in oxidative stress that may affect other diseases. Related with this, the *CDKN2B-AS1* and *CDKN2A* genes have not only been associated with POAG risk, but also with cancer and many other diseases that involve other tissues [64,65,66,67]. A possible relationship between the *CDKN2B-AS1*/*CDKN2A* genes and oxidative stress has also been reported [68]. Regarding the *TMCO1* gene, although its function seems less pleiotropic than the previous mentioned genes, there are works that relate it with several pathologies beyond those of its expression at the ocular level [69,70]. Hence, it cannot be discarded that the polymorphisms in the genes studied, apart from their direct influence on the ocular pathology, could also have a more or less indirect relationship with oxidative stress and plasma concentrations of vitamin C and E at the systemic level.

## 5. Conclusions

In our study, performed on a southern European Mediterranean population, we have detected statistically significant associations among some of the most relevant POAG polymorphisms (involving genes *TMCO1*, *CAV1*, *CAV2*, *CDKN2A* and *CDKN2B-AS1*) discovered by the initial GWASs carried out in other populations. Furthermore, we have studied them in an additive way using a GRS, showing that the risk alleles for these polymorphisms have an additive effect, with POAG risk increasing even more when they were considered simultaneously. In addition, we have found an inverse correlation between this GRS and the plasma concentrations of antioxidant vitamins (C and E), an association that remains statistically significant even after adjusting for POAG status. Although the design of our study does not allow us to show a causal association between the GRS and plasma vitamin concentrations, our results do allow us to suggest a possible connection through common mechanisms related with oxidative stress, either by the ocular or systemic pathways, an issue that will have to be investigated in greater depth in new studies.

## Figures and Tables

**Table 1:** Sociodemographic and clinical characteristics of POAG ^1^ cases and controls ^2^.

Characteristic	Cases (*n* = 391)	Controls (*n* = 383)	*p*
Females (%)	58.5	53.5	0.191
Age (years)	69.1 (9.0)	67.7 (11.1)	0.460
BMI ^3^ (kg/m^2^)	26.6 (3.8)	27.0 (4.3)	0.263
Cup disk ratio	0.69 (0.01)	0.31 (0.01)	<0.001
IOP ^4^ (mmHg)	25.3 (3.6)	16.8 (2.5)	<0.001
Smokers (%)	27.2	24.4	0.191
Alcohol consumers (%)	66.0	58.0	0.080

^1^ POAG: Primary open-angle glaucoma. ^2^ Values are means (standard deviations) for continuous variables or percentages for categorical variables. ^3^ BMI: Body mass index. ^4^ IOP: Intraocular pressure.

**Table 2:** Genotypic frequencies of the polymorphisms studied in POAG cases and controls and association with POAG risk.

Genes	SNPs	Alleles ^1^	Genotype Frequencies (%)							Genotypes			OR ^3^ (95% CI)	OR ^4^ (95% CI)
			Cases			Controls				OR ^2^ (95% CI)				
		(1/2)	1/1	1/2	2/2	1/1	1/2	2/2	*p*-Trend	1/1 (Ref)	1/2	2/2		
*TMCO1*	rs4656461	A/G	52.4	37.2	10.3	62.9	31.0	6.1	0.002	1	1.44 (1.05–1.96)	2.03 (1.17–3.52)	1.43 (1.14–1.80)	1.47 (1.16–1.86)
*CAV1*/*CAV2*	rs4236601	G/A	39.7	43.1	17.2	45.3	43.9	10.8	0.018	1	1.12 (0.82–1.52)	1.82 (1.16–2.85)	1.28 (1.04–1.58)	1.42 (1.14–1.76)
*CDKN2B-AS1*	rs2157719	A/G	29.7	45.6	24.7	37.9	43.4	18.7	0.007	1	1.34 (0.97–1.85)	1.69 (1.14–2.51)	1.30 (1.07–1.58)	1.30 (1.07–1.59)
*CDKN2A*	rs3088440	G/A	71.1	26.8	2.1	74.3	23.8	1.9	0.059	1	1.37 (0.99–1.89)	1.41 (0.52–3.84)	1.31 (0.99–1.75)	1.56 (1.15–2.12)

**^1^** Allele 1 is the major allele. Allele 2 is the minor allele. ^2^ Odds ratio (OR) and 95% confidence interval (95% CI) for each genotype (codominant model) in comparison with the reference category (homozygous subjects for the minor allele). Separate model for each polymorphism. ^3^ Global OR and 95% CI per variant allele (additive model) for each SNP obtained in the unadjusted logistic regression analysis. ^4^ Adjusted global OR and 95% CI per variant allele (additive model) for each SNP obtained in the multivariable logistic regression analysis adjusted for age and sex.

**Table 3:** Categories of the unweighted genetic risk score (uGRS) in POAG cases and controls and associations with POAG risk.

Categories ^1^ uGRS	Cases: *n* (%)	Controls: *n* (%)	OR ^2^ (95% CI)	*p ^2^*	OR ^3^ (95% CI)	*p* ^3^
Low (0 or 1)	73 (20.4)	118 (31.9)	1 (Ref)		1 (Ref)	
Medium (2 or 3)	212 (59.4)	205 (55.4)	1.67 (1.18–2.37)	0.004	1.73 (1.21–2.46)	0.002
High (4,5,6,7 or 8)	72 (20.4)	47 (12.7)	2.48 (1.54–3.96)	<0.001	2.92 (1.79–4.77)	<0.001

^1^ Three categories were considered depending on the number of risk alleles. SNPs included: *TMCO1*-rs4656461; *CAV1*/*CAV2*-rs4236601; *CDKN2B-AS1*-rs2157719; and *CDKN2A*-rs3088440. ^2^ Odds ratio (OR), 95% confidence interval (CI) and *p*-value for POAG risk depending on the category of the uGRS in the unadjusted logistic regression analysis. ^3^ Adjusted OR, 95% CI, and *p*-value for POAG risk depending on the category of the uGRS in the multivariate logistic regression analysis adjusted for age and sex.

**Table 4:** Association between the unweighted genetic risk score (uGRS) ^1^ and plasma concentrations of vitamins C and E.

Models	Regression Coefficient (B) (μg/mL per Allele)	SE	*p*
Vitamin C (μg/mL)			
Model 1 unadjusted ^2^	−0.294	0.172	<0.001
Model 2 adjusted ^3^	−0.204	0.062	0.001
Model 3 adjusted ^4^	−0.186	0.065	0.002
Vitamin E (μg/mL)			
Model 1 unadjusted ^2^	−0.248	0.180	<0.001
Model 2 adjusted ^3^	−0.205	0.067	0.002
Model 3 adjusted ^4^	−0.233	0.070	0.001

^1^ SNPs included: *TMCO1*-rs4656461; *CAV1*/*CAV2*-rs4236601; *CDKN2B-AS1*-rs2157719; and *CDKN2A*-rs3088440. ^2^ Unadjusted lineal regression model [dependent variable: plasma vitamin concentrations (C or E) as a continuous variable; independent variable: GRS as a continuous variable]. ^3^ Lineal regression model adjusted for POAG status. ^4^ Lineal regression model adjusted for POAG status, age and sex.

## Supplementary Materials

Supplementary materials can be found at www.mdpi.com/1422-0067/18/11/2302/s1.

## Abbreviations

POAG	primary open-angle glaucoma
BMI	body mass index
IOP	intraocular pressure
GWAS	genome-wide association study
GRS	genetic risk score
uGRS	unweighted genetic risk score
wGRS	weighted genetic risk score
