# Supplementary material for: A Multi-Locus Genetic Risk Score for Primary Open-Angle Glaucoma (POAG) Variants Is Associated with POAG Risk in a Mediterranean Population: Inverse Correlations with Plasma Vitamin C and E Concentrations
Source: Int J Mol Sci. 2017 Nov 1;18(11):2302. doi: 10.3390/ijms18112302 (PMC5713272; doi:10.3390/ijms18112302)
Supplement: Supplementary file 1 [file ijms-18-02302-s001.pdf]

International Journal of *Molecular Sciences*

MDPI

Supplementary Materials

A Multi-Locus Genetic Risk Score for Primary Open-Angle Glaucoma (POAG) Variants Is Associated with POAG Risk in a Mediterranean Population: Inverse Correlations with Plasma Vitamin C and E Concentrations

Vicente Zanon-Moreno, Carolina Ortega-Azorin, Eva M. Asensio-Marquez, Jose J. Garcia-Medina, Maria D Pinazo-Duran, Oscar Coltell, Jose M Ordovas, Dolores Corella.

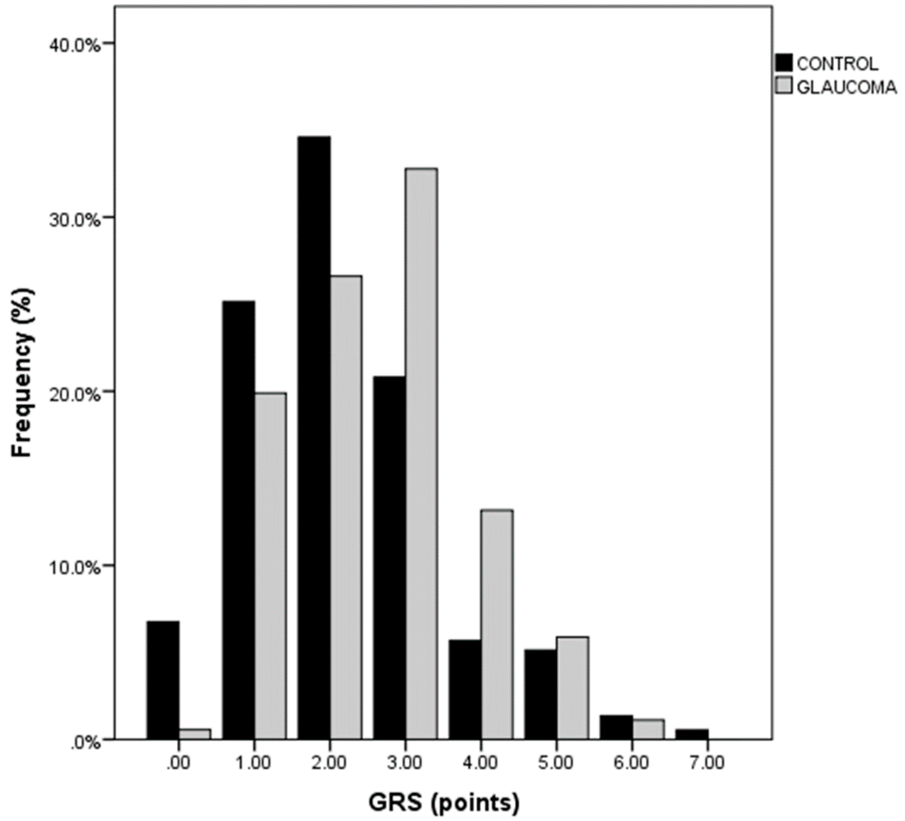

Figure S1. The unweighted GRS distribution in POAG cases and controls including the rs4656461-TMCO1, rs4236601-CAV1/CAV2, the rs2157719-CDKN2B-AS1 and the rs3088440-CDKN2A polymorphisms.
